# Eyetracking evidence for heritage speakers’ access to abstract syntactic agreement features in real-time processing

**DOI:** 10.3389/fpsyg.2022.960376

**Published:** 2022-09-30

**Authors:** Zuzanna Fuchs

**Affiliations:** Department of Linguistics, University of Southern California, Los Angeles, CA, United States

**Keywords:** heritage languages, grammatical gender, Polish, eyetracking, processing

## Abstract

This paper presents the results of an eyetracking study that uses the Visual World Paradigm to determine whether heritage speakers of Polish can use grammatical gender cues to facilitate lexical retrieval of the subsequent noun during real time processing. Previous work has investigated this question for heritage speakers of Spanish with gender cues located on definite articles, which are highly frequent in Spanish; the results are therefore consistent both with a grammatical account, wherein heritage speakers access abstract syntactic gender features during processing, and a probabilistic account, wherein facilitation is due to transition probabilities between frequently co-occurring elements. In Polish, gender cues appear on adjectives, which are optional and infrequent. Results of the present study show that heritage speakers of Polish can use gender on inflected adjectives to fixate on the target noun faster in trials where that gender cue uniquely identifies the target noun. This finding supports a grammatical rather than probabilistic account of the facilitative use of grammatical gender in this population: heritage speakers are able to access abstract syntactic information in real time to aid word recognition in a target-like manner.

## Introduction

Heritage speakers (HSs) grow up speaking and hearing a minority language at home but ultimately become dominant in the majority language spoken by the community, with a clear shift in input and dominance around school-age, when children start spending significantly less time at home, where the heritage language is spoken (Valdés, 2000; Rothman, 2009; Montrul, 2016; Kupisch and Rothman, 2018; Polinsky, 2018). As an instance of unbalanced bilingualism, heritage languages are increasingly of interest to linguists for the questions they raise regarding the impact of reduced input on the grammar and on language processing. Studies on HSs of a variety of languages have shown that in these conditions of reduced input to the heritage grammar, certain domains, such as morphosyntax, are more vulnerable and may show effects of attrition, transfer, or restructuring.

Within morphosyntax, grammatical gender has been shown to be particularly vulnerable to reduced input, with clear surface differences between heritage languages and their corresponding baseline languages, at least as evidenced by offline studies.^1^ These differences occur both in gender assignment and in gender agreement: Heritage speakers have consistently been observed to assign nouns to gender categories differently than control speakers do, and to show non-target-like comprehension and/or production of gender agreement on articles, adjectives, and/or verbs (e.g., Hindi: Montrul et al., 2012; Russian: Polinsky, 2006, 2008; Hungarian: Bolonyai, 2007; Arabic: Albirini et al., 2011, 2013; Spanish: Montrul et al., 2014, 2008; Scontras et al., 2018; Swedish: Håkansson, 1995). In fact, evidence from divergent comprehension of gender agreement suggests that surface differences in gender agreement may even be a reflex of differences in the mental representation of grammatical gender in the heritage grammar as compared to the baseline grammar (Scontras et al., 2018).

Nevertheless, recent evidence from studies using online methodologies suggests that despite surface differences in production and comprehension of grammatical gender agreement, when one controls for knowledge of gender categorization, processing of gender by HSs may be qualitatively target-like. In an eyetracking study in the Visual World Paradigm, Fuchs (2021) found that HSs of Spanish were able to fixate on target items faster when a pre-nominal gender-marked article was sufficient to uniquely identify the target item than when it was not. Fuchs concluded that HSs were able to use gender information in real-time to facilitate lexical retrieval, in a manner qualitatively like the control group. These results may suggest that early and naturalistic acquisition of gender agreement is fundamental to developing the ability to use gender to facilitate lexical retrieval (Grüter et al., 2012; Montrul et al., 2014), an idea further supported by observations of first language acquisition of nouns and articles (as discussed in more detail in section “Discussion”).

However, the finding that HSs may use gender information to comprehend nouns more efficiently warrants further investigation. Previous work on facilitative use of gender agreement in the processing of nouns in other populations has suggested that when the experimental method involves a gender cue located on an article that is frequent or obligatory in the language,^2^ such results are consistent with two possible accounts: under a syntactic account, participants are in fact accessing abstract syntactic information on the article during processing of the noun phrase; under a probabilistic account, the results reflect a mechanism that relies on surface probabilities between frequently co-occurring article-noun pairs (van Heugten and Shi, 2009; Lew-Williams and Fernald, 2010; Melançon and Shi, 2015). Existing work on HSs in this domain (Fuchs, 2021) is consistent with either account, and therefore further work is needed to adjudicate between the accounts. Under a syntactic account, we should expect to observe HSs’ facilitative use of grammatical gender when the gender cue is located on a non-frequent, non-obligatory element within the nominal phrase. Under the probabilistic account, however, we might expect significant differences between heritage and control groups in a task that provides the gender cue on such an element.

The present paper presents an eye-tracking study in the Visual World Paradigm that tests whether HSs of Polish are able to use gender information on prenominal adjectives to facilitate lexical retrieval of the subsequent noun. In existing work, this methodology has been used extensively to investigate the processing of grammatical gender by monolingual children and adults (Lew-Williams and Fernald, 2007, 2010; van Heugten and Shi, 2009; Loerts et al., 2013; Melançon and Shi, 2015, among others), as well as by L2 and—more recently—heritage bilinguals (Lew-Williams and Fernald, 2010; Grüter et al., 2012; Dussias et al., 2013; Hopp, 2013, 2016; Sekerina, 2015; Lemmerth and Hopp, 2019; Fuchs, 2021, among others). Moreover, the nominal structure of Polish is best suited for these research purposes, as the language does not have overt articles and places adjectives prenominally in the unmarked word order. The results of this study suggest that HSs of Polish are also target-like in their processing of grammatical gender, which is in-line with the earlier findings regarding HSs of Spanish. The results therefore provide additional support for early naturalistic acquisition as instrumental in developing the ability to use gender to facilitate lexical retrieval in adulthood, but call into question hypotheses regarding the exact mechanism that leads to this ability, as articles—often assumed to be central to this process—do not exist in Polish.

## Background

### Grammatical gender in heritage languages

Grammatical gender is known to be one of the more vulnerable elements of the grammar of HSs. Robust evidence from many different heritage languages suggests that HSs are non-target like with respect to gender both in their production and in their comprehension (Håkansson, 1995; Montrul et al., 2008; Polinsky, 2008; Scontras et al., 2018). This is the case despite the fact that in most languages for which this has been observed, cues to gender are reliably available in the input in the form of agreement on articles, adjectives, and/or verbs. In fact, monolingual children are able to make use of these cues to acquire fairly target-like gender agreement by around age 2–3, and monolingual adults make virtually no errors in gender agreement in naturalistic speech production (e.g., Hernández Pina, 1984; Soler, 1984; Pérez-Pereira, 1991; Mariscal, 1996, 2001; Lleó, 1997; López-Ornat, 1997). For unbalanced bilinguals, however, this is not the case; although their acquisition of gender may be roughly on par with their monolingual peers in the early stages of acquisition (Pérez-Pereira, 1991; Mariscal, 1996; Lleó, 1997; López-Ornat, 1997; Mueller Gathercole, 2002; Kuchenbrandt, 2005; Eichler et al., 2013; Ticio Quesada, 2018), they appear to diverge around 1st or 2nd grade with higher error rates in their production of gender agreement (e.g., Mueller Gathercole, 2002). Various work has found that HS children tend to over-extend the default gender more so than do their monolingual peers (Sanchez-Sadek et al., 1975; Anderson, 1999; Montrul and Potowski, 2007; Cuza and Pérez-Tattam, 2015). These patterns persist into adulthood, and manifest in divergent production and comprehension of gender agreement, as mentioned above (Montrul et al., 2008; Alarcón, 2011; Boers et al., 2020; Hur et al., 2020). Although a substantial portion of the literature on gender in heritage languages has focused on the heritage gender system in the environment of a dominant language that lacks grammatical gender (i.e., English; cf. Scontras and Putnam, 2020), work on heritage gender systems in the environment of a dominant language with grammatical gender suggests that the effect of the dominant gender system is modest if at all present: HSs whose dominant language has gender are still consistently non-target-like in their production and comprehension of gender in the HL (Cornips and Hulk, 2008; van der Linden and Hulk, 2009; Brehmer and Rothweiler, 2012; Eichler et al., 2013; Schwartz et al., 2015; Meir et al., 2017; Egger et al., 2018; Kaltsa et al., 2019; Rodina et al., 2020).

However, a growing body of evidence suggests that task modality may play a role in HSs’ performance on various experimental tasks that have been used to assess their knowledge of grammatical gender (Alarcón, 2011; Montrul et al., 2014), and online methods may be advantageous in providing a more nuanced understanding of HSs’ knowledge (Polinsky, 2018; Bayram et al., 2020; Fuchs, 2021). An example of work using online methods in this domain controls for *what* HSs know about grammatical gender in the HL and instead focuses on *how* they use that information in real time, demonstrating that HSs’ processing of grammatical gender may be target-like, counter to expectations based on HSs’ divergent performance on offline tasks. In an eye-tracking task in the Visual World Paradigm (Tanenhaus et al., 1995). Fuchs (2021) found that HSs of Spanish were able to access and deploy gender information on pre-nominal gender-marked articles to facilitate the processing of the subsequent noun. In the study, when viewing a display with two images representing lexical items of different genders (“mismatch” condition) and hearing a prompt that included a prenominal gender-marked cue (the masculine article *el* or the feminine article *la*), both HSs and the control group fixated on the target item faster than when viewing a display with two images of the same grammatical gender (“match” condition), for which the prenominal gender cue did not disambiguate the target item. Despite an absolute difference between the HSs and the controls in looking times across conditions, the HSs’ faster looks to target items in the mismatch conditions were an indication that HSs can use gender information on the gender-marked article to narrow the search in the lexicon, thus facilitating lexical retrieval, in the same manner as control speakers of Spanish.

These findings mirror patterns in previous work on monolingual speakers of Spanish (Lew-Williams and Fernald, 2007, 2010; Grüter et al., 2012; Dussias et al., 2013), German (Hopp, 2013, 2016; Hopp and Lemmerth, 2016), and Dutch (Loerts et al., 2013), among others, as well as for child speakers of Spanish (Lew-Williams and Fernald, 2007), German (Lemmerth and Hopp, 2019), and French (van Heugten and Shi, 2009; Melançon and Shi, 2015). Also notable is the fact that the findings for the HSs contrast with findings for L2 learners of languages with grammatical gender, where the findings appear to be variable with respect to whether L2 learners can also use grammatical gender during real-time processing in this way, and whether or not this ability is modulated by proficiency in the L2 (Lew-Williams and Fernald, 2010; Grüter et al., 2012; Dussias et al., 2013; Hopp, 2013; Hopp and Lemmerth, 2016). HSs and L2 learners alike fall on a spectrum of proficiency in their non-dominant language, to which they have less input than to the dominant language, resulting in non-target-like gender categorization and gender agreement for both groups. Given this, HSs patterning with adult and child controls in their ability to use gender to facilitate lexical retrieval has implications for the understanding of how the nature of the acquisition process may impact processing abilities. Following Grüter et al. (2012) and Montrul et al. (2014), Fuchs (2021) suggests that early and naturalistic acquisition of grammatical gender in the speech stream may be crucial for developing robust associations between pre-nominal gender cues and subsequent nouns, as discussed further in section “Discussion.”

However, there are outstanding questions with respect to the findings for HSs reported in Fuchs (2021). In that study, the HSs—much like the children and adults in Lew-Williams and Fernald (2007, 2010) and Grüter et al. (2012)—were prompted by auditory stimuli in which the gender cue was on a prenominal definite article. In Spanish, these articles are remarkably frequent in the input, as bare nominals are quite constrained in their distribution (cf. Mariscal, 2009; Paolieri et al., 2010). Acquirers, therefore, learn very early on that this cue to gender is both reliable and frequent. This has implications for the results of studies—whether targeting monolingual, child, or bilingual populations—investigating the facilitative use of grammatical gender specifically when the predictive gender cue is located on definite articles. What is interpreted as facilitative use of grammatical gender may on the one hand indeed be driven by a syntactic mechanism, by which participants access abstract gender agreement information in real time and use this to narrow their search within the lexicon to those items that match that gender information. However, these same results would also be consistent with a probabilistic account. Given the frequency of article-noun sequences, participants in these studies might by treating them as memorized phrases and using transitional probabilities between a given article and the candidate nouns in the VWP (Lew-Williams and Fernald, 2007, 2010; van Heugten and Shi, 2009; Melançon and Shi, 2015). This might be particularly likely for children and for HSs, who have accumulated less input in the language and are more likely to treat article-noun sequences units as unanalyzed chunks, similar to what was discussed above.

For monolingual adults and children, follow-up studies have been run to test this question, using methods that primarily involve manipulating the locus of the gender cue. That adult control speakers of Spanish are able to access gender information in real time was shown, among others, by Lew-Williams and Fernald (2010). In Experiments 2 and 3, participants learned novel nouns preceded by an indefinite gender-marked article, but were tested on those nouns using a definite gender-marked article. To succeed on the task, participants had to generalize from the information they were given in the learning phase, rather than just memorize article-noun sequences from the input. Crucially, L2 learners of Spanish in the same task were not able to generalize, suggesting that what appeared to be facilitative use of gender on articles in the initial task was driven by access to probabilistic rather than syntactic knowledge. The control group, however, was able to use gender to facilitate lexical retrieval in this version of the study, suggesting monolingual speakers do indeed access syntactic information on definite articles in real time. Testing use of gender cues on agreeing elements other than articles gets at the same issue from another approach. For instance, Hopp and Lemmerth (2016) showed that adult control speakers of German were able to use gender information on pre-nominal adjectives to facilitate lexical retrieval. Adjectives are always optional and therefore far less frequent in the input; this makes it significantly less likely that they can be treated as memorized phrases in the experimental setting and aid faster word recognition via a probabilistic process. The syntactic vs. probabilistic account has also been tested for children’s facilitative use of grammatical gender, with results in support of the syntactic account: Melançon and Shi (2015) trained 30-month-old French-speaking children on novel nouns by presenting them with gender-marked determiners and agreeing adjectives. In the testing session, they found that the children’s comprehension of the nouns was facilitated by the presence of correctly gender-marked articles, suggesting the children had generalized abstract gender information for the nouns and that they accessed this information during processing.

Given results in favor of a syntactic account for monolingual adults and children, it remains to be seen whether HSs can indeed access abstract gender information to facilitate word recognition in real time, or whether their use of gender cues on prenominal elements relies on probabilistic knowledge, more in line with the L2 learners in Lew-Williams and Fernald (2010, Experiment 2). To test this, the present study investigates whether HSs of Polish—a Slavic language that, unlike Spanish, has adjectives that appear prenominally in the unmarked word order—can use grammatical gender information on adjectives to facilitate lexical retrieval of the subsequent noun. Relevant properties of the gender system and of gender agreement marking in Polish will be introduced in the next section before detailing the research question and predictions in section “Research question.”

### Gender in Polish

Polish is generally considered to have three (global) genders—masculine, feminine, neuter—as there is evidence for three gender categories in the citation form in the nominative singular, as illustrated in (1). There are some subcategories within the masculine based on animacy, and while there has been some debate concerning the status of these subcategories, formal analyses of Polish as having three global genders and possible subgenders of masculine (Corbett, 1983) are the most widely accepted (for an overview, see Swan, 2015), and are assumed by existing work on the acquisition of Polish gender by monolingual and bilingual children (Brehmer and Rothweiler, 2012).

**Table:** 

(1)			
	a.	ta koszula	“this shirt, fem.”
		ta książka	“this book, fem.”
	b.	to jajko	“this egg, neut.”
		to okno	“this window, neut.”
	c.	ten stół	“this table, masc.”
		ten wazon	“this vase, masc.”

As illustrated in (1), each of these three genders has morphological correlates on nouns: *-a* for feminine (ex. *książka* “book, fem.”); –o, –e, or ę for neuter (ex. *okno* “window, neut.,” *imię* “name, neut.”), and consonants for masculine (ex. *stół* “table, masc.,” *talerz* “plate, masc.”). Like in most gender systems, these correspondences are not one-to-one, and there are exceptions in each gender category: *rzecz* “thing, fem.,” *coś* “something, neut.,” *mȩżczyzna* “man, masc.” Given that nominal morphophonology does not uniquely determine the gender category of a noun, the most reliable cue to grammatical gender, as generally established in formal work in this domain, is the agreement patterns that a noun determines on “associated words” in the nominal phrase (Hockett, 1958). In Polish, agreement is pervasive within the noun phrase, as gender category—as well as number and case—determine inflectional marking on attributive adjectives, relative pronouns, and demonstratives, as well as outside of the nominal phrase on predicative adjectives and verbs in certain tenses (Swan, 2015) (2). The default gender agreement for inanimate nouns in Polish is neuter, as evidenced by inflectional morphology in instances with no referent (3a) or with a genderless nominal (3b)—environments in which gender information is either absent or underspecified and therefore default gender agreement rules are deployed (Corbett and Fraser, 1999; Haspelmath, 2006).

**Table:** 

(2)					
	a.	Ten	star-y	wazon	
		DEM.M.SG	old-M.SG	vase.M	
		był	w	kuchni.	
		be.PST.3SG.M	in	kitchen	
		“That old vase was in the kitchen.”			

	b.	Ta	star-a	ksia̧żka	
		DEM.F.SG	old-F.SG	book.F	
		była	w	kuchni.	
		be.PST.3SG.F	in	kitchen	
		“That old book was in the kitchen.”			

	c.	To	star-e	wiadro	
		DEM.N.SG	old-N.SG	bucket.N	
		było	w	kuchni.	
		be.PST.3SG.N	in	kitchen	
		“That old bucket was in the kitchen.”			
(3)					
	a.	Było	zimn-o.		
		be.PST.3SG.N	cold-N.SG		
		“(It) was cold (outside).”			

	b.	[Że	Jaś	nie	przeczytał
		COMP	Jaś	NEG	read.PST.3SG.M
		lektury]	było	jasn-e.	
		book	be.PST.3SG.N	clear-N.SG	
		“That Jaś had not read the school book was clear.”			

Although this study is restricted to the nominative singular, the remainder of this section will introduce other elements of the agreement paradigm in order to provide a fuller picture of the Polish nominal agreement system. As mentioned above, the inflection on elements agreeing with the head noun is determined not only by grammatical gender but also by number (singular or plural) as well as case. Polish has six syntactic cases—nominative, genitive, dative, accusative, instrumental, and locative—as well as a seventh case (vocative) that is generally considered to be extra-syntactic and even often replaced by the nominative by modern speakers of Polish (Luczynski, 2002). The full inflectional paradigm for the adjective *stary* “old” is presented for illustration in Table 1.

**TABLE 1:** Inflectional paradigm of three global (inanimate) genders in the singular and plural of six cases.

	Singular			Plural		
	M	F	N	M	F	N
Nominative	stary	stara	stare	stare	stare	stare
Genitive	starego	starej	starego	starych	starych	starych
Dative	staremu	starej	staremu	starym	starym	starym
Accusative	stary	starą	stare	stare	stare	stare
Instrumental	starym	starą	starym	starymi	starymi	starymi
Locative	starym	starej	starym	starych	starych	starych

There are two things to note about this paradigm with respect to syncretism. First, the three genders are collapsed in the plural, making it impossible for plural agreement endings to distinguish between genders.^3^ Second, the masculine and the neuter are syncretic in the singular for all but the nominative and accusative cases. In other words, in the singular, the inflectional endings for the feminine gender are always unique from the other genders, but the masculine and the neuter are only distinguishable from each other in the nominative and accusative.

In the study presented below and in existing work on the acquisition of grammatical gender in Polish, the empirical domain has been narrowed to focus on nouns in the nominative singular. This is guided by the fact that, as mentioned above, this is one of the few parts of the inflectional paradigm where the three genders are both equally morphologically specified and unique from each other. It should be noted that unlike in other Slavic languages such as Russian, these inflectional endings do not undergo vowel reduction and are therefore reliably transparent cues to gender, a fact that may also be relevant to acquisition (Janssen, 2016). In addition, the present study investigates only gender as it occurs on inanimate nouns. Animate nouns occur in each of the three gender categories, although only within the masculine gender category does animacy determine (minimally) different inflectional paradigms. For example, animate masculine nouns take the inflectional ending *-a* in the accusative singular where all other masculine nouns take -∅; animate personal nouns take a unique inflectional ending in the nominative plural. Such minimal differences motivate some analyses of Polish gender to posit subgenders of the masculine (Corbett, 1983).^4^ Given this, the present study restricts the empirical domain to inanimate nouns.

From an acquisitional perspective, the inflectional paradigms for nominal agreement form a critical part of the input to the learner in acquiring the gender system. Recall that, although there are morphophonological correlates to gender on nouns, these are correlations rather than reliable cues with one-to-one mappings between form and gender. Whereas in languages like Spanish this lack of reliable morphophonological cues on the noun is compensated for by the consistent presence of reliable cues on gender-inflected determiners (masculine *el* and feminine *la*) that are often cited as central to the acquisition of gender classes (e.g., Mariscal, 2009; for discussion, see Fuchs et al., in press), Polish lacks such obligatory elements. Thus, the marking on adjectives is one of the main reliable cues to the acquisition of gender for Polish children. However, adjectives are optional and therefore infrequent in the input to the learner (e.g., Behrens, 2006; Stolt et al., 2008; Tribushinina and Gillis, 2012; Tribushinina et al., 2014); the gender cues that they provide are thereby also not frequent in the input. Despite the relative scarcity of gender cues in Polish, work on L1 acquisition of gender distinctions suggests that children nevertheless acquire initial distinctions by around age 2;0 (Smoczynska, 1985), similar to what has been found for languages like Spanish (Hernández Pina, 1984; Soler, 1984; Pérez-Pereira, 1991; Mariscal, 1996, 2001; Lleó, 1997; López-Ornat, 1997). However, children’s production of gender agreement suggests that the distinction between masculine and feminine may be acquired before the neuter (Dabrowska, 2006; Janssen, 2016). Krajewski (2005) argues based on evidence from a corpus of child speech (ages 1;7–2;6) that children first distinguish between the three global genders and only subsequently make animacy distinctions within the masculine gender. Although it has been proposed that diminutivization of nouns—which employs morphological marking on the noun that is consistently transparent for gender category—may also aid in the acquisition of gender by Polish children (Dabrowska, 2006), Janssen (2014) found that diminutives are less frequent in corpora of child-directed speech than previously assumed: diminutives constituted 23% of nouns in the corpus prepared by Haman et al. (2011).

Bilingual acquisition of Polish gender has been less studied. Brehmer and Rothweiler (2012) conducted a production study of bilingual Polish-German children (2;11–6;5) and found that the children produced target-like agreement marking on adjectives agreeing with masculine and feminine nouns, but overextended the masculine in producing agreement with neuter nouns. These patterns were amplified in the children’s production of agreement with nonce nouns designed to have morphophonological cues to gender consistent with the correlates of each gender category. More broadly, Haman et al. (2017) found that HSs of Polish aged 4;0–7;5 diverged from monolingual Polish speaking children in both vocabulary and grammatical knowledge, though the difference was more pronounced in production than comprehension. This is generally consistent with work that suggests HSs go through the same developmental stages in the acquisition of grammatical gender as do their monolingual peers, though with some delays (Sanchez-Sadek et al., 1975; Snyder et al., 2001; Kupisch et al., 2002; Mueller Gathercole, 2002; Kuchenbrandt, 2005; Eichler et al., 2013; Ticio Quesada, 2018).

### Research question

Existing work on grammatical gender in HLs has shown that, while adult HSs do show non-target-like knowledge of gender assignment as well as non-target-like production and comprehension of gender agreement in offline studies, their processing of gender agreement may be target-like: HSs of Spanish are faster to recognize a noun when it is preceded by a disambiguating gender-marked article (Fuchs, 2021). However, given the distribution and nature of the definite article in languages like Spanish, these findings are in fact compatible with both a grammatical account of facilitative use of grammatical gender—wherein participants access and integrate abstract gender features in real time word recognition—and a probabilistic account—wherein participants rely on transfer probabilities between article and noun derived from the frequency of the co-occurrence of these elements in the input. To test whether HSs can indeed access abstract gender information to facilitate lexical retrieval, the present study tests HSs of a language in which articles do not exist and in which pre-nominal gender cues appear on optional and infrequent elements (adjectives). The research question asked and addressed by the present study is therefore the following:

Research question: Can HSs of Polish use grammatical gender information on prenominal adjectives to facilitate the lexical retrieval of the subsequent noun?

The two conditions for comparison are the mismatch condition—in which the items in the display are of different genders, and the gender marking on the adjective may therefore serve as a facilitative cue—and the match condition—in which the items are of the same gender, and therefore the onset of the lexical item is the first unique cue to the target item. Under the syntactic account of facilitative use of grammatical gender, HSs can access the abstract syntactic gender agreement feature during online processing and use it to narrow the list of candidates in the mental lexicon, and we should therefore expect the HSs of Polish recruited for this study to fixate faster on target items in mismatch condition trials than in match condition trials—in line with what was observed for HSs of Spanish using gender information on articles to facilitate lexical retrieval in Fuchs (2021). If, however, the probabilistic account is more accurate, then we should expect HSs to be unable to use gender cues on adjectives to facilitate lexical retrieval; in this study, this means we would expect their looks to the target item to occur at about the same time across trials in both conditions.

## Materials and methods

### Materials and design

Images of 36 picturable concrete items were selected to build visual displays for the study, equally split by gender: 12 masculine, 12 feminine, 12 neuter. Corresponding lexical items were at least two syllables long to allow for looking time. To ensure a clear word boundary between the gender cue on the prenominal adjective and the onset of the noun, all lexical items had a consonant as their first phoneme. A full list of target items is available in Supplementary material. Within each gender category, 4 items were colored green, 4 red, and 4 blue. These colors were chosen because the corresponding color adjectives have an equal number of syllables. See Table 2 for the appropriately inflected forms of each color adjective.

**TABLE 2:** Three color adjectives with equal number of syllables were selected for the study.

	M	F	N
Red	czerwon-y	czerwon-a	czerwon-e
Green	zielon-y	zielon-a	zielon-e
Blue	niebiesk-i	niebiesk-a	niebiesk-ie

**FIGURE 1:**
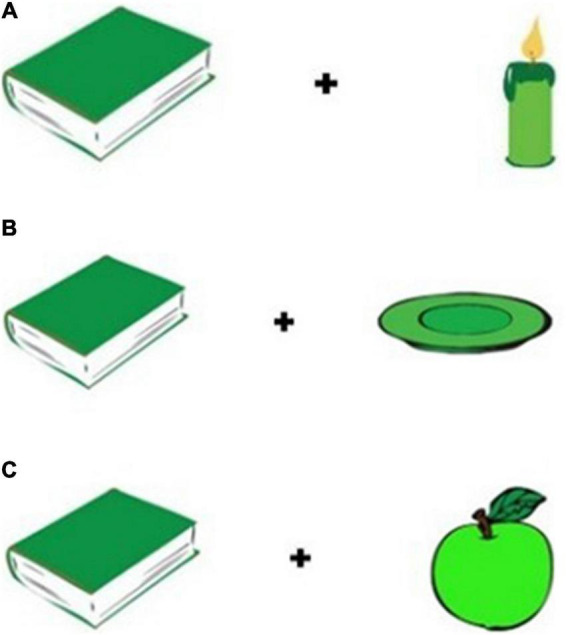
**(A)** Sample Match display with F target: *ksia̧żka* “book, fem.” and *świeczka* “candle, fem.” **(B)** Sample Mismatch-M display with F target: *ksia̧żka* “book, fem.” and *talerz* “plate, masc.” **(C)** Sample Mismatch-N display with F target: *ksia̧żka* “book, fem.” and *jabłko* “apple, neut”.

The images were combined into 108 visual displays. Each display consisted of two images equidistant from a center fixation cross; one image was the target item, the other was the distractor. Because there are three genders, for each gender there was a match condition and two mismatch conditions based on the gender of the distractor, as schematized in Table 3. Each image appeared as the target item three times: once in a match condition, and once in each mismatch condition. In total there were 36 match displays, 24 mismatch-M displays, 24 mismatch-F displays, and 24 mismatch-N displays. Sample visual displays are presented in Figure 1.

**TABLE 3:** Experimental conditions for each target gender.

		Distractor gender		
		M	F	N
	**M**	match	mismatch-F	mismatch-N
**Target gender**	**F**	mismatch-M	match	mismatch-N
	**N**	mismatch-M	mismatch-F	match

Visual displays were paired with auditory stimuli of the form in (4), prompting the participant to direct their gaze to the target item. Given that Polish does not have overt articles and that the goal was to establish whether HSs can use gender information on adjectives to facilitate lexical retrieval, the gender cue was the inflectional suffix on a color adjective (Sekerina, 2015; Hopp and Lemmerth, 2016; Lemmerth and Hopp, 2019). The overall structural simplicity of the sentence and light semantic load of the cue-carrying element were modeled after previous work on facilitative use of gender (Lew-Williams and Fernald, 2007, 2010; Grüter et al., 2012; Loerts et al., 2013; Sekerina, 2015; Hopp and Lemmerth, 2016; Lemmerth and Hopp, 2019; Fuchs, 2021).

**Table:** 

(4)	Gdzie	jest	COLOR-GEN	NOUN?
	where	is	color-GEN	noun
“Where is (the) green/red/blue noun?”				

All sentences were first recorded by a male native speaker of Polish (L2 English) immigrated to the US within 1 year of the date of recording. The final auditory prompts were created by splicing a single token of *gdzie jest* “where is” with single tokens of each of the nine inflected forms of adjectives (cf. Table 2) and tokens of each lexical item. Splicing was intended to (a) eliminate possible effects of co-articulation that might give unintended cues to the target item, and (b) to ensure that the gender cue and the lexical item occurred at the same time across stimuli for ease of comparison and analysis. For all stimuli, the onset of the gender cue occurred at 1150 ms after the start of the auditory prompt, and the onset of the lexical item occurred 480 ms later. The average duration of lexical items was approximately 700 ms.

Visual and auditory stimuli were presented together, and each trial lasted 6 s. Each trial included 800 ms of looking time and an auditory signal that prompted participants to direct their gaze to the fixation cross before the auditory stimulus began at 1000 ms into the trial. Between each trial there was a 1-s break during which only the fixation cross was visible on the screen.

### Participants

Fifty-five speakers of Polish participated in the study. Participants completed an abbreviated version of the Language Experience and Proficiency Questionnaire (LEAP-Q; Marian et al., 2007; Kaushanskaya et al., 2020) to gather demographic information and self-reported proficiency measures. The LEAP-Q was also translated into Polish for the purposes of this study,^5^ and participants could choose to fill out the LEAP-Q in either English or Polish. Control speakers of Polish were identified as those who were born in Poland and lived at least 18 years in Poland (*n* = 23). HSs of Polish were those who reported (a) that Polish was either their sole first language or their first language acquired simultaneously with English and (b) that they had lived in Poland for 8 years or less (*n* = 18).^6^

A subset of the demographic data collected from the LEAP-Q is presented in Table 4. Self-reported proficiency scores were collected, but, given the generally accepted lack of reliability of self-reported scores particularly for HSs, an oral lexical identification task was used to assess proficiency (Polinsky, 1997, 2006; Godson, 2003; Fuchs, 2021), discussed further in Section “Oral production task”.

**TABLE 4:** Selected demographic information of the control and HS participants, as self-reported in the LEAP-Q.

			**Time spent in Polish-speaking environment, in years (sd)**			
	
	***n***	**Age**	**Country**	**Family**	**Work/School**	

Controls	23	31.8 (8.7)	25.0 (8.7)	24.2 (10.8)	19.8 (9.7)	

Heritage	18	26.1 (9.9)	0.8 (1.0)	14.4 (12.5)	1.7 (3.1)	

	**Number of participants at each educational level**					
	**H.S.**	**Some coll.**	**College**	**Some Grad**	**Masters**	**PhD**

Controls	1	2	3	0	9	8

Heritage	2	3	8	1	3	1

### Procedure

Participants were tested individually in a lab. They completed the LEAP-Q either in English or in Polish, then completed the oral production task used for data cleaning and as a proxy measure for proficiency. During this task, participants viewed a set of slides with each of the 36 images used as target items during the study. They were asked to orally label each image using a color adjective and a noun. In the event that a participant was unable to recall a word for a given image, they were allowed to move on to the next image without providing a response (these responses were marked as incorrect in both coding schemes discussed in section “Results”). Their response times for each individual image were not recorded, but the total time to complete the task was recorded. This occurred prior to the comprehension task in order to assess lexical knowledge prior to exposure to the lexical items in the comprehension task (Lieberman et al., 2018; Fuchs, 2021).

Participants then completed the eye-tracking comprehension task. Participants received oral and written instructions. They sat facing a 53.5-cm screen approximately 75 cm away from it, with their head in a chin-support apparatus that ensured minimal head movement during the task. Participants saw four practice trials, after which an SMR Eyelink 1,000 was calibrated, with the goal of achieving visual acuity below 0.5 degrees. Gaze position was recorded at 2,000 Hz. The task was split into two equal parts of 54 trials each; between the two parts, participants were given a break of self-determined duration. Calibration of the eye-tracker was repeated before the second half of the task. Participation in the entire study took approximately 45 min, and participants were compensated for their time.

## Results

### Oral production task

**FIGURE 2:**
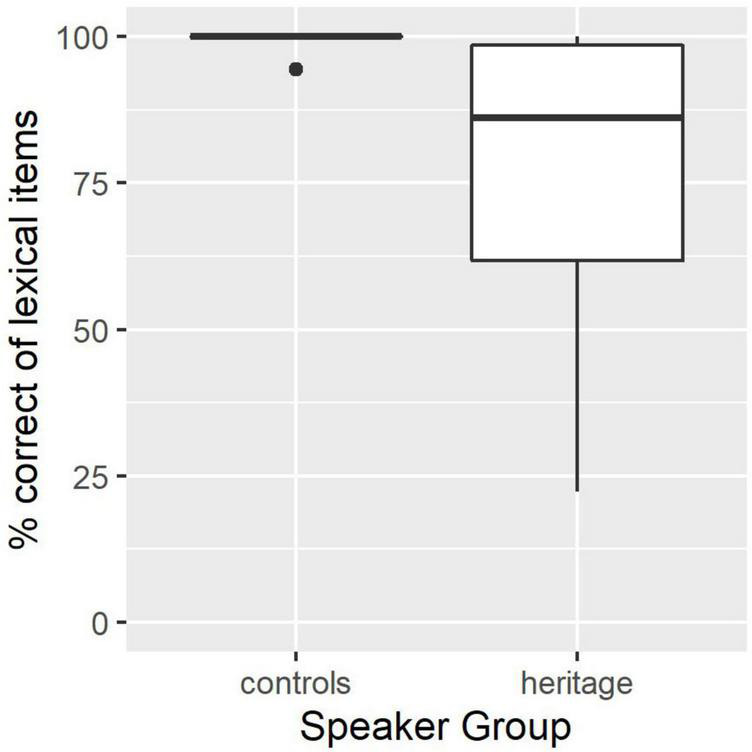
Percent of target items that participants in control and heritage groups labeled using an appropriate noun and a gender-marked adjective matching the gender of the noun.

Responses to the oral production task were coded twice, once for the purposes of a measure of lexical proficiency, and once for the purposes of data cleaning (see section “Eyetracking comprehension task”). To obtain a measure of lexical proficiency, participant responses were marked as correct if they produced an appropriate label for a given image (variants not intended by the experimental design were accepted, ex. *paczka* “package, fem.” for the intended *pudełko* “box, neut.”) along with a correctly gender-marked color adjective. The resulting proportion of correctly labeled items (out of 36) is reported in Figure 2. Control participants performed effectively at ceiling, with one or two exceptions. The HSs were able to correctly label on average approximately 28 (out of 36, sd = 8.8) of the images. There was a significant difference between the two groups (Wilcoxon Rank-Sum test, *p* < 0.001), and the HSs showed more variability (min. = 8, max. = 36).

### Eyetracking comprehension task

The aim of the study was to observe use of grammatical gender while controlling for categorization, i.e., for those lexical items for which the HSs arguably know the correct grammatical gender. To achieve this, only trials for which participants knew both the lexical items in the corresponding visual display, along with their grammatical genders, were included for analysis. This required a second coding of the oral production task: in this version, responses were coded as correct only when participants labeled a given image using the label intended by the experimental design and that label’s corresponding grammatical gender (i.e., in this case, *paczka* “package, fem.” for the intended *pudełko* “box, neut.” coded as incorrect, which is especially important given that the produced lexical item belongs to a different grammatical gender than the one intended in the experiment). Removing—for each participant—trials in which they did not label or mislabeled one or both of the images excluded 40% of the trials for the HSs and 6% of the trials for the control group.^7^ For the remaining trials, time of first fixation (response time) was gathered for each participant and analyzed in R (R Core Team, 2022) using the lme4 package (Bates et al., 2015). For linear mixed effects models, *p*-values were approximated using the Satterthwaite method implemented in the lmerTest package (Kuznetsova et al., 2017). Time of first fixation was defined as the earliest fixation on the interest area of the target item after the onset of the gender-marked adjectival suffix, which was 3250 ms after the start of each trial. The resulting times were trimmed to within two standard deviations of the mean, excluding approximately 4.6% of the data. Given the 3 × 3 experimental design, for ease of exposition the results will be presented according to the gender of the target item.

#### Feminine target noun results

**FIGURE 3:**
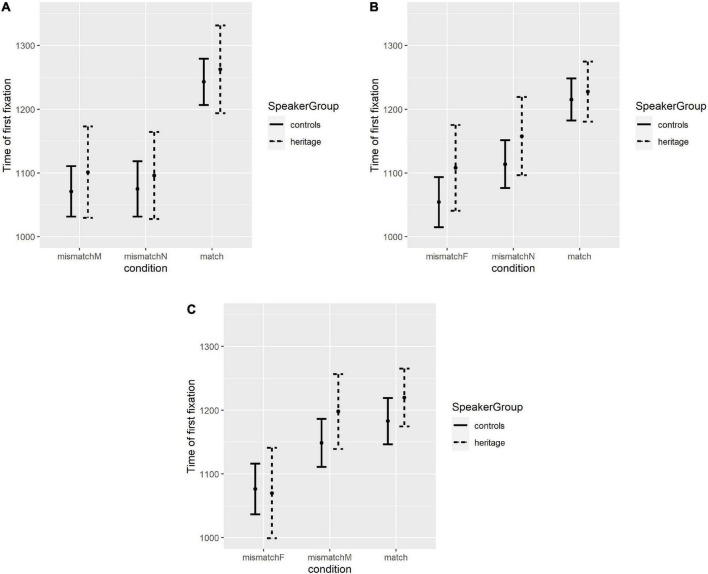
(A) Mean response times for conditions with F target items, split by group. Bars represent standard error. (B) Mean response times for conditions with M target items, split by group. Bars represent standard error. (C) Mean response times for conditions with N target items, split by group. Bars represent standard error.

The mean first fixation times for the heritage group and the control group on trials with feminine target items are presented in Figure 3A. A mixed effects linear model was fit to the data, predicting time of first fixation by GROUP, CONDITION, and TRIAL, as well as their pairwise and three-way interactions, with random intercepts and slopes for CONDITION grouped by PARTICIPANT.^8^ The categorical CONDITION variable was Helmert contrast-coded to test for a significant difference between the two mismatch conditions (for ease of exposition this is referred to as Condition-Distractor below), and then to compare the match condition to the two mismatch conditions taken together (referred to as Condition-Match below). GROUP was a categorical variable with two levels and was contrast-coded. Since the order of trials was randomized for each participant, TRIAL was a continuous variable indicating the order in which a given stimulus occurred in the study for the given participant; the variable was centered and scaled.

The model found no significant effect of Condition-Distractor (β = 2.33, SE = 12.51, *t* = 0.19, *p* = 0.852), but did find a significant effect of Condition-Match (β = 59.80, SE = 9.71, *t* = 6.16, *p* < 0.001)—participants’ mean time of first fixation was overall faster on mismatch conditions than on the match condition. The model also found a significant effect of TRIAL (β = –47.41, SE = 8.84, *t* = –5.36, *p* < 0.001): the average time of first fixation was faster in later trials. Crucially, the model did not find a significant difference between the heritage group and the control group (β = 59.48, SE = 71.77, *t* = 0.83, *p* = 0.412). A full summary of fixed effects for the model is reported in Table 5. *Post hoc* models were fit to the data to probe the significant three-way interaction between GROUP, CONDITION-DISTRACTOR, and TRIAL in the original model. For the control group, the follow-up analysis revealed no significant interaction of CONDITION-DISTRACTOR and TRIAL. The follow-up analysis for the HSs did find a significant interaction, driven by a significant effect of TRIAL on response time on Mismatch-M trials (β = –74.83, SE = 27.45, *t* = –2.73, *p* = 0.008). This indicates that, over the course of the experiment, HSs were increasingly quick to fixate on the target item in trials in which the target was feminine and the distractor was masculine.

**TABLE 5:** Fixed effects of the linear model fit to data for trials in which the target item was feminine.

	Reaction time			
	β	SE	*t*	*P*
Group	59.48	71.77	0.83	0.412
Condition-Distractor	2.33	12.51	0.19	0.852
Condition-Match	59.80	9.71	6.16	<0.001***
Trial	–47.41	8.84	–5.36	<0.001***
Group: Condition-Distractor	8.36	25.01	0.33	0.738
Group: Condition-Match	3.94	19.42	0.20	0.840
Group: Trial	–11.92	17.68	–0.67	0.500
Condition-Distractor: Trial	15.27	10.57	1.44	0.149
Condition-Match: Trial	10.23	6.35	1.61	0.107
Group: Condition-Distractor: Trial	42.18	21.14	2.00	0.046*
Group: Condition-Match: Trial	–5.94	12.70	–0.47	0.640
Constant	4406.72	35.89	122.79	<0.001***

Observations	1046			
Akaike Inf. Crit.	14604.86			
Bayesian Inf. Crit.	14684.11			

*p < 0.05; **p < 0.01; and ***p < 0.001.

#### Masculine target noun results

The mean first fixation times for the heritage group and the control group on trials with masculine target items are presented in Figure 3B. A mixed effects linear model was fit to data, predicting time of first fixation by GROUP, CONDITION, and TRIAL, as well as their pairwise and three-way interaction, with random intercepts and slopes for CONDITION grouped by PARTICIPANT and random intercepts and slopes for CONDITION grouped by adjective COLOR. The CONDITION variable was Helmert contrast-coded to test for the same contrasts as before. The full results for the fixed effects of the model are presented in Table 6. The model found a significant difference between mismatch conditions with different distractor genders (β = 27.33, SE = 11.93, *t* = 2.29, *p* = 0.032), suggesting time of first fixation on masculine target items was faster when the distractor was feminine than when the distractor was neuter. The model also found a significant difference between the match condition and the two mismatch conditions taken together (β = 38.36, SE = 9.43, *t* = 4.07, *p* = 0.003). The model also found a significant effect of TRIAL (β = –48.72, SE = 7.14, *t* = –6.84, *p* < 0.001), but no significant effect of GROUP was identified (β = 54.68, SE = 66.25, *t* = 0.83, *p* = 0.414).

**TABLE 6:** Fixed effects of the linear model fit to the data for trials in which the target item was masculine.

	Reaction time			
	β	SE	*t*	*p*
Group	54.68	66.25	0.83	0.414
Condition-Distractor	27.33	11.93	2.29	0.032*
Condition-Match	38.36	9.43	4.07	0.003**
Trial	–48.82	7.14	–6.84	<0.001***
Group: Condition-Distractor	–5.16	21.90	–0.24	0.814
Group: Condition-Match	–12.75	16.32	–0.78	0.438
Group: Trial	–12.02	14.28	–0.84	0.400
Condition-Distractor: Trial	10.44	8.75	1.19	0.233
Condition-Match: Trial	6.76	5.09	1.33	0.184
Group: Condition-Distractor: Trial	4.87	17.45	0.28	0.780
Group: Condition-Match: Trial	9.18	10.16	0.90	0.366
Constant	1155.05	34.51	33.47	<0.001***

Observations	1162			
Akaike Inf. Crit.	15975.2			
Bayesian Inf. Crit.	16071.3			

*p < 0.05; **p < 0.01; and ***p < 0.001.

#### Neuter target noun results

The mean first fixation times for the heritage group and the control group on trials with neuter target items are presented in Figure 3C. A mixed effects linear model was fit to data, predicting time of first fixation by GROUP, CONDITION, and TRIAL, as well as their pairwise and three-way interaction, with random intercepts and slopes for CONDITION grouped by PARTICIPANT and random intercepts and slopes for CONDITION grouped by adjective COLOR. The categorical CONDITION variable was Helmert contrast-coded as before. The output of this model for fixed effects is presented in Table 7. The model found a significant effect of the gender of the distractor (β = 50.78, SE = 10.21, *t* = 4.97, *p* < 0.001) as well as a significant effect of match vs. mismatch condition (β = 26.39, SE = 7.24, *t* = 3.65, *p* < 0.001). The model also found a significant effect of TRIAL (β = –40.13, SE = 7.16, *t* = –5.60, *p* < 0.001) but no significant effect of GROUP (β = 47.42, SE = 69.43, *t* = 0.68, *p* = 0.499).

**TABLE 7:** Fixed effects of the linear model fit to data for trials in which the target item was neuter.

	Reaction time			
	β	SE	*t*	*p*
Group	47.42	69.43	0.68	0.499
Condition-Distractor	50.78	10.21	4.97	<0.001
Condition-Match	26.39	7.24	3.65	<0.001
Trial	–40.13	7.16	–5.60	<0.001
Group: Condition-Distractor	25.62	20.43	1.25	0.212
Group: Condition-Match	6.80	14.48	0.47	0.641
Group: Trial	4.12	14.33	0.29	0.774
Condition-Distractor: Trial	2.52	8.58	0.29	0.769
Condition-Match: Trial	6.25	5.23	1.19	0.232
Group: Condition-Distractor: Trial	–4.68	17.16	–0.27	0.785
Group: Condition-Match: Trial	15.86	10.47	1.52	0.130
Constant	1154.21	34.72	33.25	<0.001

Observations	1116			
Akaike Inf. Crit.	15306.5			
Bayesian Inf. Crit.	15391.8			

*p < 0.05; **p < 0.01; and ***p < 0.001.

Subsequent visual analysis of Figure 3C motivated further questions regarding the contrasts between the two mismatch conditions with neuter targets. An additional *post hoc* linear mixed effects model was therefore fitted the data predicting time of first fixation by GROUP, CONDITION, and TRIAL, as well as their pairwise and three-way interactions, with random intercepts and slopes for CONDITION grouped by PARTICIPANT and random intercepts for CONDITION grouped by adjective COLOR^9^; this time, CONDITION was Helmert contrast-coded to first test for a difference between Match and Mismatch-M, and then to test for a difference between these two conditions combined as compared to the Mismatch-F condition. The model found no significant difference between Match and Mismatch-M (β = –15.71, SE = 10.03, *t* = –1.56, *p* = 0.12), but did find a significant difference between Mismatch-F and the other two conditions (β = –38.98, SE = 7.51, *t* = –5.19, *p* < 0.001). The model did not find a significant effect of GROUP (β = 47.96, SE = 69.49, *t* = 0.69, *p* = 0.49) but did find a significant effect of TRIAL (β = –40.16, SE = 7.16, *t* = 5.61, *p* < 0.001), consistent with the results of the earlier planned analysis. The output for fixed effects of this model is presented in Table 8.

**TABLE 8:** Fixed effects of the *post hoc* linear model fit to data for trials in which the target item was neuter, with CONDITION Helmert-contrast-coded to test for a difference between Mismatch-M and Match, and then between those and Mismatch-F.

	Reaction time			
	β	SE	*t*	*p*
Group	47.96	69.49	0.69	0.494
Condition-Match-Mismatch-M	–15.71	10.03	–1.56	0.120
Condition-Mismatch-F	–38.98	7.51	–5.19	<0.001***
Trial	–40.16	7.16	–5.61	<0.001***
Group: Condition-Distractor	0.61	20.07	0.03	0.976
Group: Condition-Match	–15.72	15.02	–1.04	0.302
Group: Trial	3.36	14.32	0.23	0.815
Condition-Distractor: Trial	–7.60	8.67	–0.88	0.381
Condition-Match: Trial	–4.13	5.17	–0.80	0.425
Group: Condition-Distractor: Trial	–24.81	17.35	–1.43	0.253
Group: Condition-Match: Trial	–5.82	10.35	–0.56	0.574
Constant	1154.56	34.76	33.21	<0.001***

Observations	1116			
Akaike Inf. Crit.	15305.7			
Bayesian Inf. Crit.	15391.0			

*p < 0.05; **p < 0.01; and ***p < 0.001.

## Discussion

The goal of this study was to address the following research question: Can heritage speakers of Polish use grammatical gender information on prenominal adjectives to facilitate the lexical retrieval of the subsequent noun? The prediction based on previous work on the facilitative use of grammatical gender was that, if HSs of Polish are able to use gender information to facilitate lexical retrieval, they should be able to fixate on target nouns faster in mismatch conditions, in which the agreement marking on the pre-nominal adjective provides a disambiguating cue to the subsequent noun, than on match conditions, in which the onset of the lexical item is the first available disambiguating cue. The results of the experimental study described above are consistent with this prediction: for target nouns in each of the three grammatical gender categories, participants in both groups fixated on target items faster in the mismatch conditions than, on average, in the match conditions. This indicates that, when knowledge of gender categorization is controlled for, HSs are able to access gender information in real-time and to use it to narrow the search in the mental lexicon to facilitate lexical retrieval of the target item.

The results lend support for the argument that HSs’ processing of grammatical gender in real-time is qualitatively target-like (Fuchs, 2021). The results suggest only one quantitative difference between the HSs and the control group: in conditions with feminine target nouns, HSs’ speed in fixating on the target item increased over the course of the study, whereas for the control group there was no evidence of change over time. This is indicative of a learning effect specific to the HSs, and is also consistent with previous findings for heritage speakers (Fuchs, 2021).

It is also worth noting that in some cases it appears that use of grammatical gender to facilitate lexical retrieval may be modulated by the gender of the distractor. For conditions in which the target noun was masculine, looks to the target item were slower when the distractor was neuter than when the distractor was feminine. For conditions in which the target noun was neuter, looks to the target were slower when the distractor was masculine than when the distractor was feminine—in fact, results suggest that when the distractor was masculine, the speed of first fixation on the neuter target was comparable to that of first fixation on the target item in the corresponding match condition, in which no disambiguating cue was available on the adjective. Notably, no such asymmetries between the gender of the distractor were evident when the target item was feminine.

While a thorough analysis of these patterns is beyond the scope of the present paper, these patterns suggest a hierarchical organization of the abstract gender features that leads to interference in access between some of them but not others (Fuchs, manuscript under revision). For present purposes, it suffices to say that the results suggest that these patterns are replicated in the heritage population, indicating that whatever drives the modulation of processing of the target gender by the distractor gender in Polish is equally active in the heritage grammar.

Both in the overall facilitation effect and in the modulation effect, then, the HSs in this study performed qualitatively like the control group. This indicates that, despite surface differences in gender categorization and production of gender agreement, HSs’ real-time processing of gender agreement is target-like. Combined with the results from Fuchs (2021), this underscores two important things. First, these findings echo the importance of online studies in achieving a more complete understanding of HSs’ language abilities. The view from offline studies is only partial: yes, HSs across heritage languages consistently diverge from comparison groups in gender categorization and gender agreement, but online studies now demonstrate that when one controls for gender categorization, HSs are able to access gender agreement features in real-time and integrate them into their word recognition process much like monolingual-speaking adults and children. The granularity of methods such as eye-tracking thus allows researchers to observe how speakers process linguistic information moment-by-moment, which adds critical nuance to our existing understanding of HSs’ linguistic abilities.

Second, these findings are consistent with existing proposals for early and naturalistic experience with gender agreement in nominal phrases in the speech stream as central to developing target-like processing of agreement in adulthood. While both L2 learners and HSs show divergent production and comprehension of gender agreement in offline tasks, results from VWP studies suggest HSs are able to access abstract syntactic information to facilitate lexical retrieval, similar to what has been shown for monolingual adults and children (Lew-Williams and Fernald, 2007, 2010; van Heugten and Shi, 2009; Grüter et al., 2012; Dussias et al., 2013; Hopp, 2013, 2016; Loerts et al., 2013; Melançon and Shi, 2015; Hopp and Lemmerth, 2016; Lemmerth and Hopp, 2019). This suggests that HSs’ real-time processing of gender agreement within the nominal phrase is more target-like than that of L2 learners.^10^

The explanation that has been offered for this lies in the nature of the L1 vs. the L2 acquisition processes (Lew-Williams and Fernald, 2010; Grüter et al., 2012; Fuchs, 2021). The logic is as follows: children acquire a language naturalistically from the speech stream. They encounter article-noun sequences in the input frequently, and it is thought that in an early stage of the acquisition process they treat these sequences as unanalyzed chunks, only subsequently segmenting them into an article and a noun (MacWhinney, 1978; Carroll, 1989; Pine and Lieven, 1997; Tomasello, 2000; Abu-Akel et al., 2004; Bassano et al., 2008; Mariscal, 2009). Evidence for this comes from children’s early (age 1;6–2;0) production of “proto-determiners” on nouns—pre-nominal vowels whose phonology approximates the vowel of the correct definite article (López-Ornat, 1997). It has been suggested that this acquisition process facilitates the development of a tight link between the article and noun. By contrast, L2 learners’ acquisition (at least in a traditional classroom setting), proceeds primarily from written material and is aided by a wealth of metalinguistic information, and may therefore not lead to the same robust associations between articles and nouns as in naturalistic acquisition. HSs, having acquired their heritage language in the home as children, share a naturalistic acquisition process with the L1 child and adult populations that have been investigated in these studies. Per this hypothesis, this is why HSs—despite having non-target-like agreement production and comprehension like L2 learners—nevertheless pattern with monolingual adults and children in the processing of gender in noun phrases. Montrul et al. (2014) put forward a similar hypothesis to explain why HSs performed more like the control group than the L2 learners in an offline task targeting implicit knowledge of grammatical gender.

The present results are in line with the general observation: HSs pattern with monolingual adults and children in the processing of grammatical gender, which may point to the nature of the acquisition process as being instrumental in the development of the ability to use gender agreement to facilitate lexical retrieval. However, recall that Polish does not have overt determiners—no equivalent element to the *el/la* that is overt and obligatory in most contexts for Spanish (and the equivalent for French, Italian, etc.). Therefore, the finding that HSs’ processing of gender agreement generalizes to agreement on (optional and infrequent in the input) adjectives suggests that our understanding of *what* exactly in the acquisition process is critical to the development of target-like processing of grammatical gender should also generalize beyond languages with obligatory articles.

Adjectival gender agreement has been far less studied than agreement on articles; the available existing evidence suggests it is learned later than agreement with determiners (Mariscal, 2009; Boloh and Ibernon, 2010). For children acquiring Polish naturalistically, acquiring gender agreement on adjectives poses additional challenges. While the unmarked word order is for adjectives to precede nouns in the noun phrase, adjectives may also follow the noun. Moreover, adjectives need not appear with an overt noun or even be linearly adjacent to the noun (a construction known as split nominals). Such long-distance dependencies are known to be harder to acquire than short-distance ones (Wilson et al., 2020). Polish-speaking children—whether on their way to becoming monolingual or bilingual adults—therefore have to learn grammatical gender from infrequent cues with irregular linear relationships to their target nouns.

Returning then to HSs’ target-like performance on facilitative use of grammatical gender—now observed both in Spanish and in Polish: that the HSs pattern with baseline children and adults in these studies is still evidence that the nature of the L1 acquisition process, as opposed to L2 acquisition, may play an important role in determining target-like processing of grammatical gender agreement. However, Polish demonstrates that this need not be solely linked to the cooccurrence of article-noun sequences in the input to the acquirer, whether mono- or multilingual; rather, early and naturalistic acquisition likely entails generalizing gender information from other nominal elements in the speech stream such as adjectives as well, and one of the outcomes of this acquisition is an ability to access gender information in real time processing in adulthood that is robust to pressure from reduced input to the heritage language.

## Conclusion

This paper presented the results of an eyetracking study using the Visual World Paradigm to assess the ability of heritage speakers of Polish to use gender agreement cues on prenominal adjectives to facilitate the lexical retrieval of the subsequent noun. The results showed that both HSs and the control group were able to fixate faster on target items in mismatch conditions, when the adjective inflected for gender served as a cue to the target item, than in match conditions, when the earliest disambiguating cue was the onset of the lexical item.

A previous study in this domain (Fuchs, 2021) similarly found that HSs of Spanish can use gender cues on prenominal articles to facilitate the lexical retrieval of the subsequent noun. However, the frequent and obligatory nature of these articles suggests that the results were in fact compatible with two accounts of what drove the facilitative effect. Under a syntactic account, the HSs accessed abstract gender agreement information on the pre-nominal element and integrated this information into word recognition; under a probabilistic account, HSs were not accessing gender information so much as relying on surface probabilistic properties of article-noun sequences. While the former has been found to be the case for monolingual children and adults (van Heugten and Shi, 2009; Lew-Williams and Fernald, 2010; Melançon and Shi, 2015), the latter has been found to be true for another group of unbalanced bilinguals: L2 learners (Lew-Williams and Fernald, 2010). The present paper tested these two accounts of processing of gender agreement in the noun phrase for HSs by using eye-tracking in the VWP to determine whether HSs of Polish can use gender information on inflected pre-nominal adjectives to facilitate lexical access of the subsequent noun.

The results indicate that HSs can indeed access and deploy abstract gender agreement information on pre-nominal elements during real-time processing in a target-like manner. Taken together with previous work on facilitative use of grammatical gender in monolingual and bilingual populations, these findings have implications for our understanding of what determines target-like processing of grammatical gender in adulthood. Although HSs are like L2 learners in their generally observed non-target-like gender categorization and gender agreement, they nevertheless pattern with monolingual adults and children in their facilitative use of grammatical gender. The results are consistent with the proposal that early and naturalistic acquisition of grammatical gender from the speech stream is likely central to the development of this ability (Lew-Williams and Fernald, 2010; Grüter et al., 2012; Montrul et al., 2014; Fuchs, 2021), as it captures why HSs, L1 children, and baseline adults pattern together to the exclusion of adult L2 learners. However, it calls into question the assumption that it is precisely the frequent co-occurrence of articles and nouns that is central to the development of target-like processing of gender agreement in the noun phrase—this proposal is simply untenable for languages like Polish, which do not have overt articles and whose flexible word order implies gender cues do not necessarily appear linearly adjacent to nouns in the input. Nevertheless, the finding that HSs of both Spanish and Polish can use gender information on agreeing elements to facilitate the lexical retrieval of nouns in real time suggests that this ability is robust to reduced input to the heritage grammar.

## Data availability statement

The raw data supporting the conclusions of this article will be made available by the author, without undue reservation.

## Ethics statement

The studies involving human participants were reviewed and approved by the University of Maryland IRB. The participants provided their written informed consent to participate in this study.

## Author contributions

ZF designed and conducted the study, completed the statistical analysis, and wrote the manuscript.

## Footnotes

1 The terms “baseline” and “control” are used herein to indicate the population of comparison, instead of terms such as “native” or “monolingual”. HSs are native speakers in their own right, by virtue of the nature of their acquisition process (for discussion see Pascual et al., 2012; Kupisch and Rothman, 2018; Polinsky, 2018, Chp. 2.3; Wiese et al., 2022).

2 While this may apply to both definite and indefinites articles, the discussion in the literature has primarily focused on definite articles, as these cooccur most frequently with nouns and are the most frequent gender cue in the input to child speakers of languages like Spanish that have gender agreement and obligatory articles (cf. Mariscal, 2009, p. 144).

3 The exception are certain subcategories of the masculine, which are restricted to animate nouns and are not discussed here. These subcategories have been the subject of debate in the formal literature on the number of Polish gender (sub)categories (see Corbett, 1983; Swan, 2015, among others, for overview and discussion).

4 Complications in how to treat animacy in this system come from both empirical and theoretical considerations. From an empirical perspective, evidence that recent loanwords pattern with traditionally animate non-human masculine nouns in their inflectional paradigm has called into question the status of animacy in the Polish gender system (Fuchs, 2014). From a theoretical perspective, there is also some debate as to whether animate gender features occur in the same locus in the nominal structure or whether they are representationally independent of inanimate gender information (Kramer, 2009, 2014; Steriopolo and Wiltschko, 2010; Bobaljik and Zocca, 2011; but see also Kramer, 2015).

5 The Polish translation of the LEAP-Q is now available at https://bilingualism.northwestern.edu/leapq/.

6 Fourteen participants were excluded from data analysis. A majority of these left Poland after the age of 8 but before 18 and therefore did not reliably belong to either category. Two reported that their L1 was neither English nor Polish.

7 These percentages of data removal are similar to the outcome of the same procedure applied in Fuchs (2021).

8 An additional model with random intercepts and slopes for CONDITION grouped by COLOR was attempted but resulted in a singular fit.

9 A similar model with added random slopes for CONDITION grouped by COLOR was attempted but resulted in a singular fit.

10 Dussias et al. (2013) present an exception, as they found that L1-Italian L2-Spanish learners could fixate on target items faster in mismatch than match conditions, but only when the target item was feminine. The authors speculate this may be driven by overlap in the gender systems of the two languages and/or by the similarity in form between feminine definite articles in the two languages.
