# Supplementary material for: Eyetracking evidence for heritage speakers’ access to abstract syntactic agreement features in real-time processing
Source: Front Psychol. 2022 Sep 30;13:960376. doi: 10.3389/fpsyg.2022.960376 (PMC9562099; doi:10.3389/fpsyg.2022.960376)
Supplement: Supplementary file 1 [file Table_1.DOCX]

Supplementary Material

# Supplementary table: Target items

|  | Item | gen. | English |
| --- | --- | --- | --- |
| 1. | drabina | f. | ladder |
| 2. | drzewo | n. | tree |
| 3. | dzbanek | m. | kettle |
| 4. | gniazdo | n. | nest |
| 5. | grzebień | m. | comb |
| 6. | huśtawka | f. | swing |
| 7. | jabłko | n. | apple |
| 8. | jajko | n. | egg |
| 9. | koło | n. | wheel |
| 10. | koszula | f. | shirt |
| 11. | krzesło | n. | chair |
| 12. | książka | f. | book |
| 13. | lalka | f. | doll |
| 14. | linijka | f. | ruler |
| 15. | lustro | n. | mirror |
| 16. | łańcuch | m. | chain |
| 17. | łopata | f. | shovel |
| 18. | łóżko | n. | bed |
| 19. | młotek | m. | hammer |
| 20. | mydło | n. | soap |
| 21. | namiot | m. | tent |
| 22. | pasek | m. | belt |
| 23. | pióro | n. | feather |
| 24. | pudło | n. | box |
| 25. | ręka | f. | hand |
| 26. | rower | m. | bicycle |
| 27. | samolot | m. | airplane |
| 28. | strzała | f. | arrow |
| 29. | sukienka | f. | dress |
| 30. | szalik | m. | scarf |
| 31. | świeczka | f. | candle |
| 32. | talerz | m. | plate |
| 33. | truskawka | f. | strawberry |
| 34. | wiadro | n. | bucket |
| 35. | widelec | m. | fork |
| 36. | zegar | m. | clock |
